# Primary Gastrointestinal T/NK Cell Lymphoma

**DOI:** 10.3390/cancers13112679

**Published:** 2021-05-29

**Authors:** Eun Kyung Kim, Mi Jang, Woo Ick Yang, Sun Och Yoon

**Affiliations:** 1 Department of Pathology, National Health Insurance Service Ilsan Hospital, Goyang 10444, Korea; dalsoon2@nhimc.or.kr (E.K.K.); mjrose@nhimc.or.kr (M.J.); 2 Department of Pathology, Severance Hospital, Yonsei University College of Medicine, Seoul 03722, Korea; wiyang9660@yuhs.ac

**Keywords:** T/NK cell lymphoma, gastrointestinal tract, intestinal lymphoma, clinicopathologic features

## Abstract

**Simple Summary:**

Primary gastrointestinal T/NK cell lymphoma (GI-TNKL) is a heterogeneous group including various subtypes, anatomic locations, and clinicopathologic features. GI-TNKL is difficult to diagnose due to its rarity, various subtypes, and histopathological features. We performed a retrospective analysis of 38 cases of GI-TNKL in South Korea. GI-TNKL consisted of unique subtypes showing specific characteristics of macroscopy, histology, immunophenotype, and prevalent anatomic subsites. ENKTL and MEITL were relatively common. GI-TNKL showed aggressive behavior with short PFS and OS. This clinical and pathological descriptive analysis will be helpful for accurate understanding, diagnosis, and treatment.

**Abstract:**

Primary gastrointestinal T/NK cell lymphoma (GI-TNKL) is an uncommon and heterogeneous group of lymphoid malignancies. We aimed to investigate their subtype distribution, clinicopathologic characteristics, and clinical outcomes. A total of 38 GI-TNKL cases and their clinical and pathological characteristics were analyzed. GI-TNKL occurred in adults with a median patient age in the sixth decade of life and showed a slight male predominance. The most common histologic type was extranodal NK/T-cell lymphoma, nasal type (ENKTL; 34.2%), followed by monomorphic epitheliotropic intestinal T-cell lymphoma (MEITL; 31.6%), intestinal T-cell lymphoma, NOS (ITCL, NOS, 18.4%), anaplastic large cell lymphoma, ALK-negative (ALCL, ALK-; 13.2%). The small intestine was the primary affected region. More than 90% of patients complained of various GI symptoms and cases with advanced Lugano stage, high IPI score, or bowel perforation that required emergent operation were not uncommon. GI-TNKL also showed aggressive behavior with short progression-free survival and overall survival. This thorough clinical and pathological descriptive analysis will be helpful for accurate understanding, diagnosis, and treatment.

## 1. Introduction

The gastrointestinal tract is the most common site of extranodal lymphomas and primary gastrointestinal lymphomas are mostly of B cell lineage. Primary gastrointestinal T/NK cell lymphoma (GI-TNKL) is generally rare, but is relatively more common in East Asia where it accounts for 13–15% of primary GI lymphoma [1,2,3,4]. GI-TNKL is a heterogeneous group including various subtypes, anatomic locations, and clinicopathologic features. The revised fourth edition of the World Health Organization (WHO) classification recently included enteropathy-associated T-cell lymphoma (EATL), monomorphic epitheliotropic intestinal T-cell lymphoma (MEITL), intestinal T-cell lymphoma, NOS (ITCL, NOS), and indolent T-cell lymphoproliferative disorder of the GI tract in the new category ‘intestinal T-cell lymphoma’ [5]. In addition, extranodal NK/T-cell lymphoma, nasal type (ENKTL) and anaplastic large cell lymphoma (ALCL) often occur in the GI tract. EATL is the most common GI-TNKL in Western countries because it occurs in individuals with celiac disease, but it is extremely rare in Asia [6]. Except for indolent T-cell lymphoproliferative disorder of the GI tract, GI-TNKL is usually aggressive and is frequently accompanied by GI complications such as bowel perforation, obstruction, and bleeding, which increase morbidity and mortality [7,8]. There have been few specific studies on T/NK cell lymphoma primarily developed in the GI tract. In this study, we performed a retrospective analysis of 38 cases in South Korea to improve the knowledge of clinicopathologic features of GI-TNKL.

## 2. Materials and Methods

### 2.1. Patient Selection and Histological Review

Primary gastrointestinal lymphoma was defined as predominant lesions in the alimentary tract with or without regional lymph node involvement [9]. We excluded post-transplant lymphoproliferative disorder and indolent T-cell lymphoproliferative disorder of the GI tract. A total of 38 primary gastrointestinal T/NK cell lymphoma (GI-TNKL) cases were retrieved from Severance Hospital and National Health Insurance Service Ilsan Hospital from January 2005 to December 2017. All hematoxylin and eosin-stained and immunostained slides were reviewed by experienced hematopathologists (S.O.Y., W.I.Y., and E.K.K.) and histologic classification was undertaken according to the revised fourth edition of the WHO classification of neoplastic disease of hematopoietic and lymphoid tissue [5]. Cases showing the histology of peripheral T cell lymphoma, not otherwise specified (PTCL, NOS), which primarily involves the GI tract but could not be categorized as MEITL or other T/NK cell lymphoma subtypes, were defined as intestinal T cell lymphoma, not otherwise specified (ITCL, NOS). Clinical information and survival data including serum lactate dehydrogenase (LDH), β-2 microglobulin (B2M), Eastern Cooperative Oncology Group (ECOG) performance status, international prognostic index (IPI), and findings of imaging studies and endoscopy were obtained by review of medical records. Patients were staged according to the Lugano staging system for GI lymphomas [10]. Lugano stage I is defined as a tumor confined to the GI tract with a single primary site or multiple noncontiguous lesions. Stage II is a tumor that extends into the abdomen with local or distant lymph node involvement and stage IIE is defined as a tumor with adjacent organ involvement. Stage IV is defined as disseminated extranodal disease or concurrent supradiaphragmatic nodal involvement. Details for the ancillary tests are summarized in Supplementary Data.

### 2.2. Statistical Analysis

The chi-square, Fisher exact, and one-way ANOVA tests were used to analyze differences between evaluated variables. Overall survival (OS) was measured from date of initial diagnosis to date of death or last follow-up. Progression-free survival (PFS) was measured from the date of initial diagnosis to that of disease recurrence, progressive disease without complete remission, or disease-related death during the study period. The Kaplan-Meier method was used to analyze survival rates, and differences therein were compared using the log-rank test. Two-sided *p* values < 0.05 were considered statistically significant. Multivariate analysis was performed with the Cox proportional hazard model. Statistical analyses were conducted using IBM SPSS 23 software for Windows (IBM Corp., Armonk, NY, USA).

## 3. Results

### 3.1. Clinicopathologic Characteristics of Primary Gastrointestinal T/NK Cell Lymphoma

Among 836 primary GI lymphoma cases diagnosed from 2005 to 2017, mature B cell lymphomas accounted for the majority (797 cases, 95.3%). Primary GI mature B cell lymphoma mainly consisted of extranodal marginal zone lymphoma of mucosa-associated lymphoid tissue (MALT) type (399 cases, 50.1%) and diffuse large B cell lymphoma (310 cases, 38.9%). Among the primary GI lymphomas, 356 cases (43.7%) were gastric MALT lymphoma. T/NK cell lymphomas were 38 cases, accounting for 4.5% (38/836) of all primary GI lymphomas and 8.1% (38/471) when gastric MALT lymphomas were excluded.

Clinicopathologic characteristics of these patients with GI-TNKL are summarized in Table 1. The median age was 58 years (range, 34 to 84), and the male to female ratio was 1.4:1. The most common histologic type was ENKTL (*n* = 13, 34.2%), followed by MEITL (*n* = 12, 31.6%), ITCL, NOS (*n* = 7, 18.4%), ALCL, ALK- (*n* = 5, 13.2%), and ALCL, ALK+ (*n* = 1, 2.6%). The most frequently affected anatomic location was the small intestine (71.1%), followed by large intestine (34.2%), stomach (23.7%), ileocecum (13.2%), and esophagus (2.6%). Multiple site involvement was found in 31.6% of patients. Most patients had symptoms, with abdominal pain and discomfort being the most common complaint at the initial presentation, followed by weight loss and poor oral intake, diarrhea, and GI bleeding. GI complications developed in 63.2% during the disease, including perforation, hemorrhage, fistula formation, and obstruction. Lugano stage was advanced (stage IV) in 47.4% of patients and others were stage I or II. IPI score was 3 or more (high or high-intermediate) in 31.6%, and 7.9% presented ECOG performance status 2 or more.

Fifty percent of patients were diagnosed by surgical resection and nine patients (23.7%) underwent emergency surgery. Excluding five patients who could not confirm specific treatment methods and four patients who died at the time of hospitalization after the first diagnosis and surgery, 15 patients (39.5%) received both surgery and non-surgical treatment, and the remaining patients (*n* = 14, 36.8%) received non-surgical treatment as the first line therapy. Of the 29 patients who received first-line chemotherapy, four received autologous peripheral blood stem cell transplantation, and two received radiation therapy. CHOP (cyclophosphamide, doxorubicin hydrochloride, vincristine sulfate, and prednisone) with or without etoposide was the most common first-line drugs, followed by IMVP-16PL (ifosfamide, methotrexate, etoposide, cyclophosphamide, and dexamethasone; Supplementary Table S1). Eighteen patients (47.4%) died while receiving first-line treatment, or died without second-line treatment after progression or recurrence.

### 3.2. Comparison of Clinical Features of Primary Gastrointestinal T/NK Cell Lymphoma Subtypes

Comparisons of clinical features of GI-TNKL subtypes are presented in Table 2 and Supplementary Figure S1. The small intestine was the most frequent main location in ENKTL, MEITL, and ITCL, NOS (*p* = 0.002). Esophageal or gastric involvement was more common in ALCL and the least common in MEITL (*p* = 0.003). When endoscopic examination was possible or when the lesion was endoscopically visible, it appeared as a single or multiple mass, deep ulcer, superficial ulcer, or relatively normal. Mass lesion was the most common finding (Table 3). GI complication during the course of the disease appeared frequently in ENKTL and MEITL (*p* = 0.074). Perforation most frequently occurred in ENKTL, followed by MEITL (*p* = 0.026). Advanced stage was most frequent in ENKTL patients (*p* = 0.085). For initial diagnosis or treatment, ENKTL, MEITL, and ITCL, NOS were more likely to have a surgical procedure used, whereas non-surgical methods were mainly used for ALCL (*p* = 0.009). Treatment response for initial chemotherapy showed complete remission (CR) in 53.6% and progressive disease (PD) in 21.4%. ENKTL showed the highest CR rate and also PD rate (*p* = 0.564). One case, a 34-year-old male patient, had ALCL, ALK+ at the stomach and showed complete initial chemotherapy response without recurrence.

### 3.3. Morphologic and Immunophenotypic Features of Primary Gastrointestinal T/NK Cell Lymphoma Subtypes

**Figure 1:**
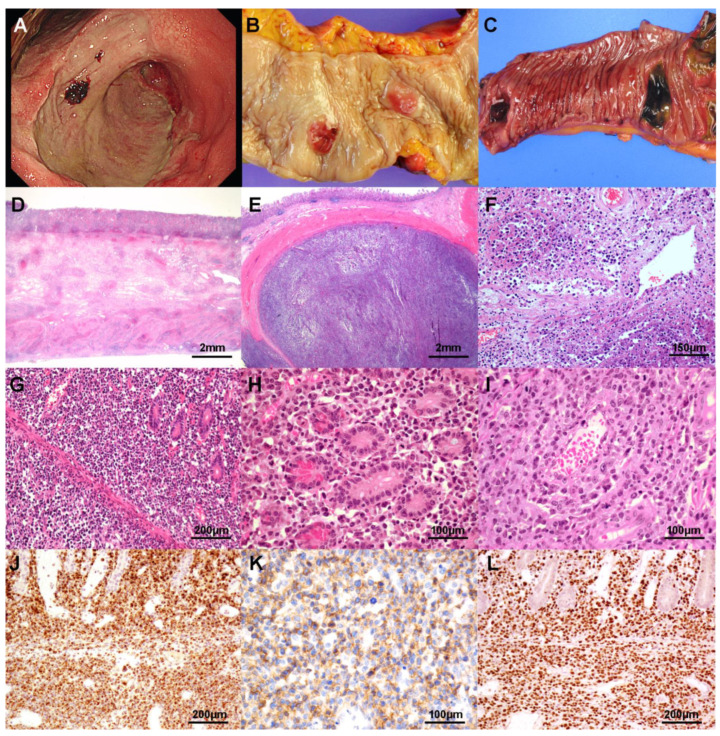
Primary gastrointestinal extranodal NK/T cell lymphoma, nasal type. Endoscopy reveals a huge ulceroinfiltrative lesion with an irregular base in the stomach (**A**). This case shows multiple hyperemic nodular lesions in the colon (**B**) and small bowel. The small bowel mucosa shows several various sized ulcerations (**C**). Microscopy shows diffuse transmural involvement (**D**) and a nodular mass in the low power view (**E**). Angiodestruction and necrosis are frequent findings (**F**). Tumor cells involve mucosa and submucosa and glands are relatively preserved (**G**). Medium to large atypical lymphoid cells infiltrate the lamina propria without epitheliotropism (**H**). Heterogeneous tumor cells show angiocentric pattern (**I**). Tumor cells are positive for granzyme B (**J**), CD56 (**K**), and EBV-encoded small RNA (**L**).

**Figure 2:**
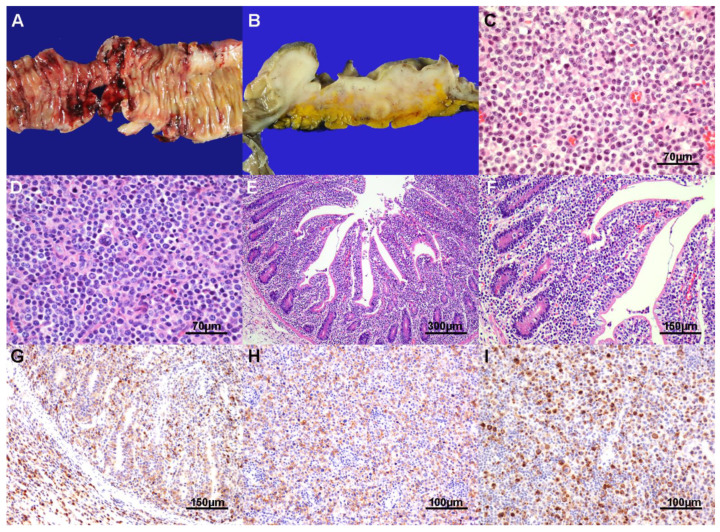
Monomorphic epitheliotropic intestinal T-cell lymphoma. The jejunum shows multiple ulceration with perforation (**A**). The transverse colon shows an ulceroinfiltrative mass involving mesenteric lymph nodes (**B**). Tumor cells are monotonous and medium-sized without inflammatory background (**C**). Some cases show variably admixed large pleomorphic cells (**D**). Tumor cells spread to the surrounding mucosa showing intraepithelial lymphocytosis (**E**) with relatively preserving villous architecture (**F**). Tumor cells are positive for CD8 (**G**), CD56 (**H**), and granzyme B (**I**).

**Figure 3:**
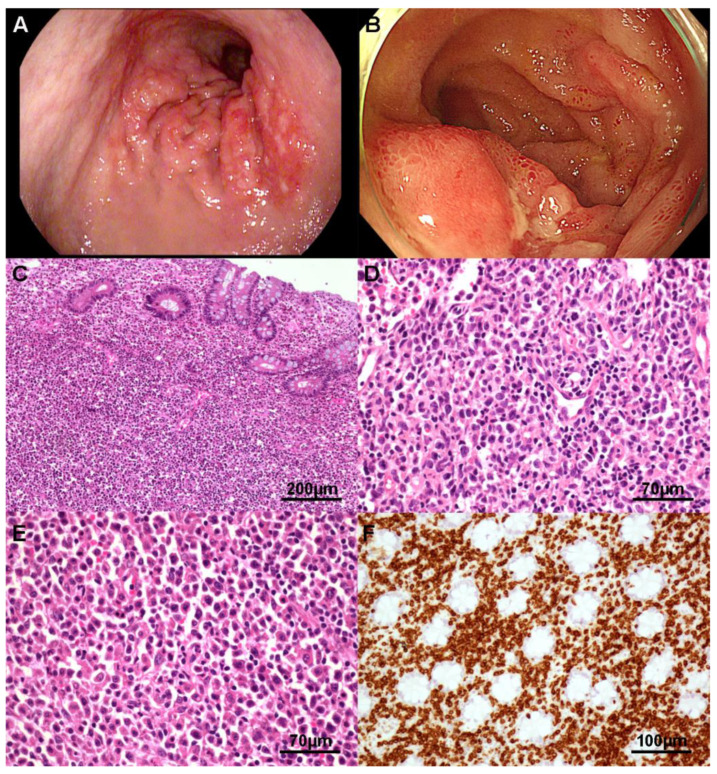
Intestinal T-cell lymphoma, not otherwise specified. Stomach endoscopy shows multiple serpiginous-shaped superficial erosions with surface nodularity (**A**). Terminal ileum endoscopy demonstrates several geographic shallow and deep ulcerations (**B**). Tumor cells show diffuse and destructive growth (**C**). Some cases show medium to large atypical lymphoid cells with inflammatory background (**D**) or large pleomorphic cells with eosinophilic cytoplasm (**F**). Tumor cells are CD3 positive and reveal no epitheliotropism (**D**).

**Figure 4:**
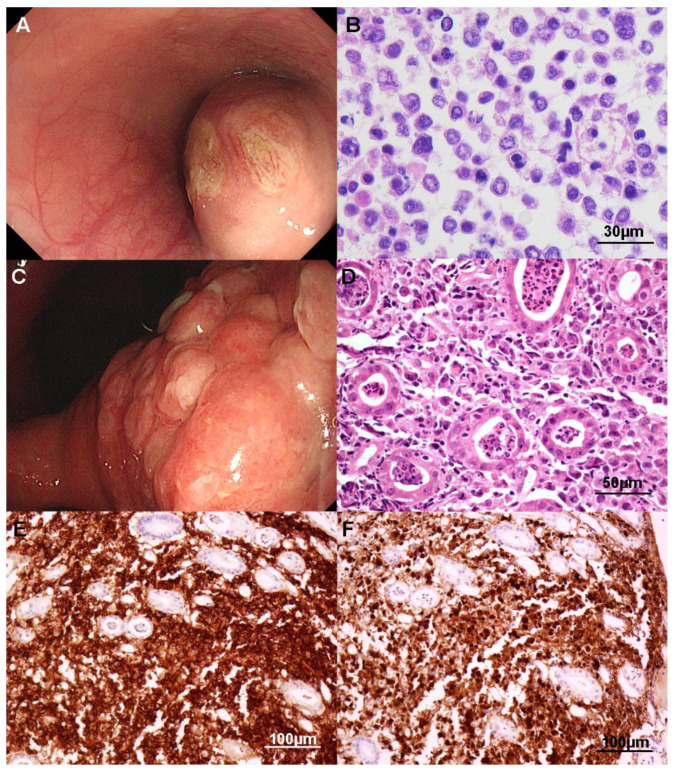
Anaplastic large cell lymphoma (ALCL). ALCL, ALK-negative (**A**,**B**). Endoscopically, the esophagus shows a large elevated submucosal tumor covered with mucosal ulceration (**A**). Biopsy findings demonstrate non-cohesive medium to large pleomorphic cells with irregular nuclei (**B**). ALCL, ALK-positive (**C**–**F**). Endoscopy reveals hyperemic irregular and nodular surface with abnormalities of surrounding folds in the stomach (**C**). The same case shows large pleomorphic lymphoid cells with eccentric nuclei and abundant cytoplasm in the neutrophilic background (**D**). The tumor cells are immunoreactive for CD30 (cytoplasmic, nuclear membrane and Golgi region; **E**) and ALK (cytoplasm and nucleus; **F**).

The morphologic and immunophenotypic features of each lymphoma type are summarized in Table 3 and Figure 1, Figure 2, Figure 3 and Figure 4. ENKTL appeared as single or multiple infiltrative masses (33.3%) or ulceration in endoscopy (66.7%; Figure 1A). In resection specimens, ENKTL grossly appeared with solitary or several ulcerative masses or deep ulcerations (Figure 1B,C). All seven resection cases involved subserosal or serosal layer infiltration by tumor cells (Figure 1D,E). Regional lymph nodes were also frequently involved (76.9%). They usually consisted of medium to large tumor cells and all cases showed heterogeneous tumor cells (Figure 1F–I). Tumor cells frequently demonstrated a prominent angiocentric and angiodestructive pattern (46.2%, Figure 1F,I), while epitheliotropism was not found (Figure 1G,H). Background was usually inflammatory or necrotic. Tumor cells were all immunoreactive for CD3, granzyme B (Figure 1J), and TIA-1; variable for CD56 (69.2%; Figure 2K). They were all reactive in EBER ISH (Figure 1L). Three out of eight patients (37.5%) who underwent the whole blood EBV quantitative PCR test were positive (Supplementary Table S2). The average proliferation index was high. A T-cell receptor (TCR) gene rearrangement study was performed in five cases and two cases had monoclonal TCR gene rearrangement.

MEITL endoscopically manifested a mass or a superficial ulcer except for cases with perforation or jejunal lesion. A case that showed relatively normal appearance in endoscopy was histologically correlated with intraepithelial lymphocytosis. Most of the cases were located in the small intestine. Multiple site involvement and perforation were frequent (Figure 2A,B). Most of the eight resected cases showed transmural involvement and regional lymph nodes were also frequently involved.

As a definition, almost all cases revealed monomorphic tumor cells (Figure 2C) with marked epitheliotropism. Large pleomorphic cells were variable admixed (Figure 2D). Villous architecture was usually distorted and broadened by tumor cells. Tumor cells commonly spread to the surrounding mucosa with intraepithelial lymphocytosis, and the villous architecture was relatively preserved (Figure 2E,F). Angiocentricity, inflammatory background, or prominent tumor necrosis was not observed. Tumor cells were positive for CD3, CD8 (72.7%; Figure 2G), CD56 (Figure 2H), CD103 (60%), granzyme B (81.8%; Figure 2I), and TIA-1 (66.7%), while negative for CD20, CD10, PD-1, ALK, and EBER ISH. Proliferation index was high. TCR gene rearrangement study was performed in five cases and all had monoclonal TCR gene rearrangement.

Most ITCL, NOS were located in the intestine. ITCL, NOS likely appeared as superficial ulcer (Figure 3A) or mass (Figure 3B), and frequently revealed transmural involvement. Tumor cells were medium to large (71.4%) and occasionally showed predominantly large pleomorphic cells. They were usually heterogeneous with angulated and hyperchromatic nuclei and a scant amount of eosinophilic cytoplasm (Figure 3C–F). Tumor cells were CD3 positive (Figure 3D) and reactive for both CD4 and CD8. They were mostly positive for granzyme B (83.3%), occasionally positive for CD30 and CD56. One case showed EBER ISH positivity in reactive B-cells, not tumor cells. Ki-67 labeling index was variable. A TCR gene rearrangement study was conducted in one case and showed TCRG clonal rearrangement.

ALCL, ALK- was mostly located in the stomach rather than the intestine and manifested as a submucosal mass (Figure 4A), an ulcerofungating mass, or deep ulcer. Tumor cells were medium to large or pleomorphic and heterogeneous. Nuclear membranes were irregular and occasionally showed hallmark (kidney-shaped) cells and multinucleated cells. Nuclear chromatin was vesicular or hyperchromatic and eosinophilic cytoplasm was scant to abundant (Figure 4B). The background was rich in small vasculature and inflammatory cells. Tumor cells were all diffusely immunoreactive for CD30 within the cytoplasm and/or at the cell membrane or in the Golgi area. They were variable for CD3, CD4, CD10, granzyme B, TIA-1, and PD-1, and negative for CD8, CD56, CD103, ALK, and EBER ISH. A case of ALCL, ALK+ in the stomach was endoscopically an irregularly shaped nodular mass (Figure 4C). The biopsy specimen revealed similar histologic features to ALCL, ALK- (Figure 4D). Tumor cells were positive for CD3 and granzyme B. CD30 was diffusely positive (Figure 4E) and ALK was also diffusely expressed in cytoplasm and nuclei (Figure 4F).

### 3.4. Survival Analysis

The median follow-up time was 10.4 months (range: 0.1–122.0 months). Twenty-nine patients (76.3%) died of disease and 32 patients (84.2%) experienced disease recurrence or progression during the follow-up period. ALCL, ALK+ cases had no recurrence or death and were excluded from survival analysis. Median OS was 9.6 months and median PFS was 5.1 months (Table 2). There were no significant differences in OS and PFS between the subtypes. ENKTL showed the lowest median OS (5.1 months) and ALCL, ALK- revealed the worst median PFS (2.8 months). In the Kaplan-Meir analysis, Lugano stage, IPI score ≥ 3, initial treatment response, and serum LDH elevation were significantly associated with OS and PFS (Supplementary Figure S2). Initial treatment response was an independent factor for OS (PD: HR 63.369, *p* < 0.001) and PFS (PD: HR 16.976, *p* = 0.001) in multivariate analysis. Elevated serum LDH level (HR 5.045, *p* = 0.015) was also an independent factor for OS (Table 4).

## 4. Discussion

Primary GI-TNKL is a heterogeneous and rare disease group, and there are few extensive clinicopathologic studies including the entire GI tract ranging from the esophagus to the large intestine. In the present study, we investigated clinicopathologic features of 38 Korean GI-TNKL cases. GI-TNKL accounted for 4.5% of all primary GI lymphomas. This figure is somewhat less than the 13–15% frequency of GI-TNKL reported in East Asia [1,2,3,4]. However, some of those studies included only the intestinal tract, unlike our study that also included the stomach and esophagus. In the large-scale study of gastrointestinal tract lymphoma conducted at an institution in Southwest China, the frequency of GI-TNKL was 12.9% [3]. In that study, gastric MALT lymphoma accounted for 15.5% of all GI lymphomas, whereas in our cohort, it was much higher at 43.7%. That is, since gastric MALT lymphoma is more common in South Korea than in other countries, the frequency of GI-TNKL may be relatively low. When gastric MALT lymphoma was excluded, the frequency of GI-TNKL rose to 8.1%.

In our cohort, GI-TNKL occurred in adults with a median patient age in the sixth decade of life and a slight male predominance. The most common histologic type was ENKTL, followed by MEITL, ITCL, NOS, ALCL, ALK-, and ALCL, ALK+. The small and large intestines were the most affected regions. More than 90% of patients complained of various GI symptoms and over half of patients suffered from GI complications. Half of patients had surgical treatment, of which 47.4% underwent emergency operation. Half of patients completely responded to initial chemotherapy, while about half of patients had relapse or progression of the disease. These frequencies and characteristics are similar to previous studies reported in the Asian region [1,2,3,11,12].

On initial endoscopic findings, 71.4% had mass lesions or deep ulceration and 23.8% showed superficial ulcers. The single MEITL case that had nearly normal appearance in endoscopy revealed intraepithelial lymphocytosis in histology. There were two other cases that had lesions in the jejunum and they were clinically diagnosed by computed tomography (CT) imaging instead of endoscopy. Since GI-TNKL, which occurs most frequently in small intestine, may not show any specific findings on the endoscope, it is crucial for early correct diagnosis to suspect this rare disease in patients complaining of GI symptoms.

About 70% (9/13) of ENKTL showed NK cell phenotype (CD56-positive). In the remaining four cases, two cases were negative for both CD4 and CD8, and the other two cases were focal positive for both CD4 and CD8. However, they were all positive for granzyme B, suggesting cytotoxic T cell phenotype. Similarly, clonal TCR gene rearrangement was observed in the two cases with T cell phenotype.

The level of circulating EBV DNA in blood is not included in the diagnostic criteria of ENKTL, and EBV infection should be confirmed in tumor cells [5]. In addition, EBV positivity in tumor cells or blood is not always the same as the diagnosis of ENKTL, and may occur in other T-cell lymphoma subtypes as well. In our cohort, EBV positivity in blood was 37.5% in ENKTL, 33.3% in MEITL, and 16.7% in ITCL (Supplementary Table S2). However, since EBV DNA can be released from tumor cells into plasma, the level of circulating EBV DNA is a reliable marker for tumor load and can be useful for monitoring disease activity and predicting prognosis. High EBV DNA titers have been reported to be associated with extensive diseases, unfavorable response to treatment, and low survival rates [13,14]. Although the number of positive cases in this study was small and EBV DNA quantification tests were performed in whole blood and not in plasma, the EBV DNA titer was at least 5 times higher in ENKTL than MEITL and ITCL. Three ENKTL cases with high EBV titer were stage IV at the time of diagnosis, and two cases with follow-up data died within 2 months of chemotherapy.

MEITL was most frequently located in the jejunum (75%). There was a case of single gastric lesion (8.3%) and a case of multiple large intestinal lesions (8.3%) without small intestinal involvement. Gastric or colonic alone MEITL is rare and has been reported at about 2.4% and 18%, respectively [15,16].

In two previous studies, aberrant expression (heterogeneous and weak pattern) of CD20 was reported in some MEITLs with frequencies of 24% and 11%, respectively [17,18]. However, no such aberrant expression of CD20 was observed in this study.

The revised fourth edition of the WHO classification of neoplastic disease of hematopoietic and lymphoid tissue has newly categorized ITCL, NOS, which arises in the intestines or sometimes other sites in the gastrointestinal tract and does not fulfill the criteria for other T/NK cell lymphomas, including MEITL or ENKTL [5]. In our institution, primary intestinal T cell lymphoma which cannot be categorized as a known entity has been diagnosed as PTCL, NOS. Most were located in the small intestine or ileocecum (71.4%) and the remainder were in the stomach. Tumor cells were CD4+/CD8+ (80%) or CD4-/CD8- (20%).

Compared to other subtypes, ALCL was more often located in the upper GI tract and GI complications were relatively less common. These patients underwent chemotherapy rather than surgery. Although four of the five ALCL, ALK- cases were Lugano stage I or II, the median OS and PFS were relatively shorter compared to other GI-TNKLs. ALCL, ALK- is known to exhibit significant clinical and genetic heterogeneity. Chromosomal rearrangements of DUSP22 or TP63 occur in about 30% and 8% of ALCL, ALK-, respectively [19]. DUSP22-rearranged ALCLs are associated with favorable prognosis similar to ALCL, ALK+, while TP63-rearranged ALCLs have very poor outcomes [19]. To predict the clinical course of ALCL, ALK-, immunohistochemistry for p63 can be a useful screening test [20]. It is highly sensitive for TP63 rearrangement but is not specific. TP63 rearrangement must be confirmed by genetic methodology such as fluorescence in situ hybridization (FISH) [20]. Unfortunately, due to the lack of adequate and available tissue, additional tests to identify genetic subtypes were not conducted in this study.

Except for one case of ALCL, ALK+, disease recurrence or progression in patients with GI-TNKL was common (84.2%). The prognosis was poor with a median survival of 9.6 months and median PFS of 5.1 months. OS and PFS showed no significant differences among the histologic subtypes as in the previous study [2]. IPI score (≥3), serum LDH or B2M elevation, and initial treatment response (PD) were associated with poor OS and PFS. Among them, elevated serum LDH and initial treatment response (CR or PD) were independent prognostic factors for OS. The initial treatment response (PR, SD, or PD) was an independent prognostic factor for PFS. Although GI complications may affect mortality, there was no statistically significant effect on prognosis. Since this study has a relatively small number of cases, there may be some limitations in survival analysis.

Recent studies have shown the characteristic genetic alterations in GI-TNKL. Whole exome and next generation sequencing of six primary GI-ENKTLs showed mutations in *JAK3* and the voltage gated potassium channels [21]. In MEITL, mutations of JAK1 and STAT5B, genes related to the JAK/STAT signaling pathway, mutations in KRAS, a gene related to the RAS signaling pathway, a loss-of-function mutation of SETD2, a chromatin modifying gene, were found [21,22,23]. There is little research on the genetic alteration of ITCL to date. In a previous study, JAK3, SETD2, and NRAS mutations have been reported in ITCL [21]. Therefore, ITCL is thought to share some similar genetic features with MEITL. Although further robust studies are required, such genetic alterations might be promising with regard to the treatment of GI-TNKL.

CHOP with or without etoposide has been most widely used regimen in our cohort. However, CHOP is no longer the standard choice for ENKTL treatment and it is less effective in MEITL as well [24]. Concurrent or sequential chemoradiation therapy is currently considered as the standard treatment for patients with localized ENKTL [25]. L-Asparaginase or pegylated asparaginase containing regimens are widely used in the treatment of advanced and relapsed/refractory ENKTL [26,27]. Since the outcomes of various treatment regimens currently used are still poor, several novel approaches targeting for PD-1/PD-L1 [28], LMP1/LMP2 [29], and JAK/STAT pathway (NCT02974647) are being conducted in ENKTL [30], and STAT5 [31,32] in MEITL. In ALCL, CHOP or CHOP plus etoposide remains the first-line therapy [33] and recent clinical trials with anti-CD30 antibody (brentuximab vedotin) have shown substantial activity in the relapsed or refractory disease [34] and front-line treatment [35]. Autologous hematopoietic stem cell transplantation as front line consolidation in PTCL, NOS, ALCL, ALK-, ENKTL (disseminated), and enteropathy-associated T-cell lymphoma is recommended by expert consensus [36].

## 5. Conclusions

Primary GI-TNKL consisted of unique subtypes showing specific characteristics of macroscopy, histology, immunophenotype, and prevalent anatomic subsites. Among them, ENKTL and MEITL were relatively common. Cases with advanced Lugano stage, high IPI score, or bowel perforation that required emergent operation were not uncommon. GI-TNKL also showed aggressive behavior with short PFS and OS. Primary GI-TNKL is difficult to diagnose due to its rarity, various subtypes, and histopathological features. A thorough clinical and pathological descriptive analysis will be helpful for accurate understanding, diagnosis, and treatment.

## Figures and Tables

**Table 1:** Clinicopathologic characteristics of 38 patients with primary gastrointestinal T/NK cell lymphoma.

Characteristics	*n* (%)
Median age (range)	58 years (34–84)
Male/Female (M:F ratio)	22/16 (1.4:1)
Subtypes of lymphoma	
ENKTL	13 (34.2)
MEITL	12 (31.6)
ITCL, NOS	7 (18.4)
ALCL, ALK-	5 (13.2)
ALCL, ALK+	1 (2.6)
Location	
Esophagus	1 (2.6)
Stomach	9 (23.7)
Small intestine	27 (71.1)
Duodenum	4 (10.5)
Jejunum	15 (39.5)
Ileum	8 (21.1)
Ileocecum	5 (13.2)
Large intestine	13 (34.2)
Ascending/transverse	6 (15.8)
Descending/sigmoid	6 (15.8)
Rectum	1 (2.6)
Multiple site involvement	12 (31.6)
Initial presentation	
Abdominal pain and discomfort	20 (52.6)
Weight loss and poor oral intake	8 (21.1)
Diarrhea	7 (18.4)
GI bleeding	5 (13.2)
Vomiting	3 (7.9)
No symptom	2 (5.3)
GI complication	24 (63.2)
Perforation	15 (39.5)
Hemorrhage	4 (10.5)
Fistula formation	3 (7.9)
Obstruction	2 (5.3)
No initial GI complication	14 (36.8)
Lugano stage	
I	8 (21)
II	12 (31.6)
IV	18 (47.4)
ECOG performance status ≥ 2	3/34 (7.9)
IPI score ≥ 3	12 (31.6)
Anemia	21 (55.3)
Lymphopenia	21 (55.3)
Thrombocytopenia	2 (5.3)
Diagnostic procedure	
Endoscopic biopsy	19 (50)
Surgery	19 (50)
First treatment	
Surgery with adjuvant therapy	15 (39.5)
Non-surgical therapy	14 (36.8)
Surgery	4 (10.5)
Unknown	5 (13.2)
First line non-surgical treatment	29
CTx alone	24 (82.8)
CTx + autoPBSCT	3 (10.3)
CTx + RTx + autoPBSCT	1 (3.5)
RTx + CTx	1 (3.4)

ALCL, ALK-; anaplastic large cell lymphoma, ALK-negative, ALCL, ALK+; anaplastic large cell lymphoma, ALK-positive, autoPBSCT; autologous peripheral blood stem cell transplantation, ECOG; Eastern Cooperative Oncology Group, ENKTL; extranodal NK/T cell lymphoma, nasal type, GI; gastrointestinal tract, IPI; International prognostic index, ITCL, NOS; intestinal T-cell lymphoma, not otherwise specified, MEITL; monomorphic epitheliotropic intestinal T-cell lymphoma, RTx; radiation therapy.

**Table 2:** Comparison of clinical features of primary gastrointestinal T/NK cell lymphoma subtypes.

Variable		Total	ENKTL	MEITL	ITCL, NOS	ALCL, ALK-	ALCL, ALK+	*p* Value
		*n* = 38 (100%)	*n* = 13 (34.2%)	*n* = 12 (31.6%)	*n* = 7 (18.4%)	*n* = 5 (13.2%)	*n* = 1 (2.6%)	
Age > 60		13 (34.2)	5 (38.5)	2 (16.7)	4 (57.1)	2 (40)	0 (0)	0.882
Mean age		58.3	58.9	55.5	62	63	34	0.297
Sex	Male	22 (57.9)	9 (69.2)	6 (50)	3 (42.9)	3 (60)	1 (100)	0.777
	Female	16 (42.1)	4 (30.8)	6 (50)	4 (57.1)	2 (40)	0 (0)	
Main-location	Esophagus	1 (2.6)	0 (0)	0 (0)	0 (0)	1 (20)	0 (0)	0.002
	Stomach	9 (23.7)	2 (15.4)	1 (8.3)	2 (28.6)	3 (60)	1 (100)	
	SI	17 (44.7)	5 (38.5)	9 (75)	3 (42.9)	1 (20)	0 (0)	
	IC	7 (18.4)	2 (15.4)	0 (0)	2 (28.6)	0 (0)	0 (0)	
	LI	4 (10.5)	4 (30.8)	2 (16.7)	0 (0)	0 (0)	0 (0)	
Esophageal/gastric involvement		10 (26.3)	2 (15.4)	1 (8.3)	2 (28.6)	4 (80)	1 (100)	0.003
Multiple site involvement		13 (34.2)	4 (30.8)	5 (41.7)	3 (42.9)	1 (20)	0 (0)	0.767
GI complication		24 (63.2)	10 (76.9)	8 (66.7)	4 (57.1)	2 (40)	0 (0)	0.074
GI perforation		15 (39.5)	8 (61.5)	5 (41.7)	1 (14.3)	1 (20)	0 (0)	0.026
Lugano stage	I/II	20 (52.6)	5 (38.5)	6 (50)	4 (57.1)	4 (80)	1 (100)	0.085
	IV	18 (47.4)	8 (61.5)	6 (50)	3 (42.9)	1 (20)	0 (0)	
Elevated serum LDH level		17/36 (42.7)	5/11 (45.5)	5 (45.5)	4 (57.1)	2 (40)	1 (100)	0.663
Elevated serum B2M level		22/30 (73.3)	7/8 (87.5)	7/11 (63.6)	4/6 (66.7)	3/4 (75)	1 (100)	0.857
IPI score ≥ 3		12 (31.6)	5 (38.5)	4 (33.3)	2 (28.6)	1 (20)	0 (0)	0.365
Initial treatment	OP ± CTx/RTx	23 (62.2)	10 (83.3)	8 (66.7)	4 (57.1)	1 (20)	0 (0)	0.009
	CTx/RTx	14 (37.8)	2 (16.7)	4 (33.3)	3 (42.9)	4 (80)	1 (100)	
CTx response	CR	15/28 (53.6)	5/8 (62.5)	5/10 (50)	3/6 (50)	1/3 (33.3)	1 (100)	0.486
	PR	6/28 (21.4)	0/8 (0)	3/10 (30)	1/6 (16.7)	2/3 (66.7)	0 (0)	
	SD	1/28 (3.6)	0/8 (0)	0/10 (0)	1/6 (16.7)	0/3 (0)	0 (0)	
	PD	5/28 (21.4)	3/8 (37.5)	2/10 (20)	1/6 (16.7)	0/3 (0)	0 (0)	
Recurrence/progression		32 (84.2)	9 (69.2)	12 (100)	6 (85.7)	5 (100)	0 (0)	0.116
Median OS (months)		9.6	5.1	11.2	16.6	9.6	-	0.829
Median PFS (months)		5.1	3.8	5.8	7.7	2.8	-	0.844

ALCL, ALK-; anaplastic large cell lymphoma, ALK-negative, ALCL, ALK+; anaplastic large cell lymphoma, ALK-positive, B2M; beta-2 microglobulin, CR; complete response, CTx; chemotherapy, ENKTL; extranodal NK/T cell lymphoma, nasal type, GI; gastrointestinal tract, IC; ileocecum, IPI; International prognostic index, ITCL, NOS; intestinal T-cell lymphoma, not otherwise specified, LDH; lactate dehydrogenase, LI; large intestine, MEITL; monomorphic epitheliotropic intestinal T-cell lymphoma, OP; operation, OS; overall survival, PD; progressive disease, PFS; progression-free survival, PR; partial response, SD; stable disease, SI; small intestine. Statistically significant values were boldfaced.

**Table 3:** Comparison of endoscopic and pathological features of primary gastrointestinal T/NK cell lymphoma subtypes.

Variable	Total	ENKTL	MEITL	ITCL, NOS	ALCL, ALK-	ALCL, ALK+
	*n* = 38	*n* = 13	*n* = 12	*n* = 7	*n* = 5	*n* = 1
Endoscopy						
Mass	10/21 (47.6)	2/6 (33.3)	2/5 (40)	2/5 (40)	3/4 (75)	1 (100)
Deep ulcer	5/21 (23.8)	3/6 (50)	0/5 (0)	1/5 (20)	1/4 (25)	0 (0)
Superficial ulcer	5/21 (23.8)	1/6 (16.7)	2/5 (40)	2/5 (40)	0/4 (0)	0 (0)
Normal	1/21 (4.8)	0/6 (0)	1/5 (20)	0/5 (0)	0/4 (0)	0 (0)
Depth of invasion						
Mucosa/submucosa	0/19 (0)	0/7 (0)	0/8 (0)	0/3 (0)	0/1 (0)	-
Muscularis propria	2/19 (10.5)	0/7 (0)	1/8 (12.5)	0/3 (0)	1/1 (100)	-
Subserosa/serosa	17/19 (89.5)	7/7 (100)	7/8 (87.5)	3/3 (100)	0/1 (0)	-
Regional lymph node involvement	24 (63.2)	10 (76.9)	6 (50)	5 (71.4)	2 (40)	1 (100)
Cell size						
Small to medium	3 (7.9)	1 (7.7)	2 (16.7)	0 (0)	0 (0)	0 (0)
Medium to large	26 (68.4)	10 (76.9)	10 (83.3)	5 (71.4)	2 (40)	0 (0)
Large pleomorphic	9 (23.7)	2 (15.4)	0 (0)	2 (28.6)	3 (60)	1 (100)
Cell shape						
Heterogeneous	24/36 (66.7)	13 (100)	0 (0)	5 (71.4)	5 (100)	1 (100)
Monomorphic	12/36 (33.3)	0 (0)	12 (100)	2 (28.6)	0 (0)	0 (0)
Angiocentricity	6/36 (16.7)	6 (46.2)	0 (0)	0 (0)	0 (0)	0 (0)
Epitheliotropism	12/36 (33.3)	0 (0)	12 (100)	0 (0)	0 (0)	0 (0)
CD3	37 (97.4)	13 (100)	12 (100)	7 (100)	4 (80)	1 (100)
CD4	9/25 (36)	2/8 (25)	1/9 (11.1)	4/5 (80)	2/3 (66.7)	-
CD8	15/26 (57.7)	3/9 (33.3)	8/11 (72.7)	4/5 (80)	0/2 (0)	-
CD10	1/15 (6.7)	0/6 (0)	0/5 (0)	0/1 (0)	1/2 (50)	0 (0)
CD30	12/33 (36.4)	4/11 (36.4)	1/8 (12.5)	1/5 (20)	5 (100)	1 (100)
CD56	21/36 (58.3)	9 (69.2)	11 (91.7)	1 (14.3)	0/4 (0)	-
CD103	6/19 (31.6)	0/5 (0)	6/10 (60)	0/2 (0)	0/2 (0)	-
Granzyme B	28/33 (50.9)	10/10 (100)	9/11 (81.8)	5/6 (83.3)	3/5 (6))	1 (100)
TIA-1	13/18 (72.2)	7/7 (100)	4/6 (66.7)	0/2 (0)	2/3 (66.7)	-
PD-1	1/15 (6.7)	0/6 (0)	0/5 (0)	0/2 (0)	1/2 (50)	-
ALK	1/16 (6.25)	0/7 (0)	0/5 (0)	0/2 (0)	0 (0)	1 (100)
Ki-67 L.I. (%, average)	70.6	70	83.3	41.7	75	100
EBER ISH	9/26 (34.6)	8/8 (100)	0/9 (0)	0/6 (0)	0/3 (0)	-
TCR gene rearrange-ment ^a^	8/11 (72.7)	2/5 (40)	5/5 (100)	1/1 (100)	-	-
*TCRG*	5/11	1/5	3/5	1/1	-	-
*TCRB*	4/11	1/5	3/5	0/1	-	-
*TCRD*	1/11	0/5	1/5	0/1	-	-

ALCL, ALK-; anaplastic large cell lymphoma, ALK-negative, ALCL, ALK+; anaplastic large cell lymphoma, ALK-positive, EBER ISH; EBV-encoded RNA in situ hybridization, ENKTL; extranodal NK/T cell lymphoma, nasal type, ITCL, NOS; intestinal T-cell lymphoma, not otherwise specified, L.I.; labeling index, MEITL; monomorphic epitheliotropic intestinal T-cell lymphoma, TCR; T-cell receptor, ^a^ BIOMED-2 based PCR analysis using IdentiClone TCRG, TCRB, and TCRD Gene Clonality Assay Kits (Invivoscribe, CA, USA).

**Table 4:** Cox proportional hazards regression model of primary gastrointestinal T/NK cell lymphoma.

	Overall Survival				Progression Free Survival			
Variables	Univariate		Multivariate		Univariate		Multivariate	
	*p* Value	HR (95% CI)	*p* Value	HR (95% CI)	*p* Value	HR (95% CI)	*p* Value	HR (95% CI)
>60 years	0.066	2.369 (0.953–4.350)	-	-	0.050	2.079 (1.001–4.318)	-	-
Male	0.728	1.141 (0.542–2.403)	-	-	0.424	1.335 (0.657–2.711)	-	-
Subtypes								
ENKTL	0.831	1 (reference)	-	-	0.846	1 (reference)	-	-
MEITL	0.453	1.429 (0.563–3.626)	-	-	0.426	1.430 (0.593–3.450)	-	-
ITCL, NOS	0.960	1.009 (0.312–3.019)	-	-	0.762	1.175 (0.414–3.340)	-	-
ALCL, ALK-	0.602	1.397 (0.439–4.135)	-	-	0.488	1.480 (0.489–4.477)	-	-
Location								
Esophagus	0.171	1 (reference)			0.383	1 (reference)		
Stomach	0.563	1.856 (0.228–15.082)	-	-	0.496	0.477 (0.057–4.025)	-	-
Small intestine	0.504	2.012 (0.259–15.606)	-	-	0.434	0.437 (0.055–3.470)	-	-
Ileocecum	0.631	0.554 (0.050–6.174)	-	-	0.125	0.145 (0.012–1.707)	-	-
Large intestine	0.601	0.544 (0.055–5.326)	-	-	0.212	0.242 (0.026–2.249)	-	-
Multiple site	0.990	1.005 (0.453–2.228)	-	-	0.930	1.034 (0.493–2.170)	-	-
UGI involvement	0.404	0.715 (0.324–1.574)	-	-	0.282	0.651 (0.297–1.424)	-	-
GI complication	0.348	1.448 (0.668–3.139)	-	-	0.112	1.854 (0.865–3.971)	-	-
GI perforation	0.219	1.598 (0.757–3.376)	-	-	0.305	1.449 (0.713–2.945)	-	-
Tx with surgery	0.798	0.906 (0.425–1.930)	-	-	0.782	1.105 (0.545–2.242)	-	-
Lugano stage IV	0.051	2.214 (0.996–4.533)	-	-	0.041	2.119 (1.031–4.354)	0.299	0.461 (0.107–1.989)
IPI ≥ 3	<0.001	4.922 (2.022–11.979)	0.485	1.570 (0.442–5.581)	0.001	4.209 (1.846–9.597)	0.391	1.686 (0.511–5.556)
Elevated LDH	0.038	2.248 (1.045–4.835)	0.015	5.045 (1.365–18.654)	0.019	2.446 (1.155–5.718)	0.076	2.903 (0.894–9.424)
Elevated B2M	0.023	3.685 (1.194–11.368)	0.429	1.835 (0.407–8.273)	0.037	2.940 (1.066–8.108)	0.827	1.160 (0.308–4.369)
Initial Tx response								
CR	0.001	1 (reference)	0.003	1 (reference)	<0.001	1 (reference)	0.005	1 (reference)
PR	0.012	5.064 (1.418–18.079)	0.058	6.374 (0.940–43.236)	0.001	10.720 (2.56–44.84)	0.043	5.911 (1.061–32.928)
SD	0.385	2.611 (0.299–22.781)	0.429	2.662(0.235–30.093)	0.002	61.45 (4.58–824.33)	0.006	45.692 (3.12–790.67)
PD	<0.001	24.554 (5.353–112.62)	<0.001	63.369 (6.60–608.41)	<0.001	24.22 (5.31–110.43)	0.001	16.976 (3.14-91.65)

ALCL, ALK-; anaplastic large cell lymphoma, ALK-negative, ALCL, ALK+; anaplastic large cell lymphoma, ALK-positive, B2M; beta-2 microglobulin, CI; confidence interval, CR; complete response, ENKTL; extranodal NK/T cell lymphoma, nasal type, GI; gastrointestinal, HR; hazard ratio, ITCL, NOS; intestinal T-cell lymphoma, not otherwise specified, MEITL; monomorphic epitheliotropic intestinal T-cell lymphoma, LDH; lactate dehydrogenase, PD; progressive disease, PR; partial response, SD; stable disease, Tx; treatment, UGI; upper gastrointestinal tract (esophagus and stomach). Statistically significant values were boldfaced.

## Data Availability

Pathology data and the statistical analyses for the current study are available from the corresponding author upon reasonable request.

## Supplementary Materials

The following are available online at https://www.mdpi.com/article/10.3390/cancers13112679/s1, Methods: Immunohistochemistry. Table S1: First-line treatment of primary gastrointestinal T/NK cell lymphoma. Table S2: The results of whole blood EBV quantitative PCR test in patient with primary gastrointestinal T/NK cell lymphoma. Figure S1: Comparison of clinical features of primary gastrointestinal T/NK cell lymphoma sub-types. Figure S2: Kaplan-Meier survival curves according to clinicopathological parameters.

## Abbreviations

ALCL: ALK-	anaplastic large cell lymphoma, ALK-negative
ALCL, ALK+	anaplastic large cell lymphoma, ALK-positive
B2M	beta-2 microglobulin
CR	complete remission
CT	computed tomography
EBER	Epstein-Barr virus-encoded RNA
EBV	Epstein-Barr virus
ECOG	Eastern Cooperative Oncology Group
ENKTL	extranodal NK/T-cell lymphoma, nasal type
FISH	fluorescence in situ hybridization
GI-TNKL	primary gastrointestinal T/NK cell lymphoma
HR	hazard ratio
IHC	immunohistochemical staining
IPI	international prognostic index
ITCL, NOS	intestinal T-cell lymphoma, NOS
LDH	lactate dehydrogenase
MALT	mucosa-associated lymphoid tissue
MEITL	monomorphic epitheliotropic intestinal T-cell lymphoma
OS	overall survival
PD	progressive disease
PFS	progression-free survival
PR	partial response
SD	stable disease
TCR	T-cell receptor
WHO	World Health Organization
