# Supplementary material for: Primary Gastrointestinal T/NK Cell Lymphoma
Source: Cancers (Basel). 2021 May 29;13(11):2679. doi: 10.3390/cancers13112679 (PMC8199162; doi:10.3390/cancers13112679)
Supplement: Supplementary file 1 [file cancers-13-02679-s001.zip › cancers-1208270-supplementary.pdf]

Supplementary Materials: Primary Gastrointestinal T/NK Cell Lymphoma

Eun-Kyung Kim, Mi Jang, Woo-Ick Yang and Sun-Och Yoon

Methods: Immunohistochemistry

Immunohistochemistry was performed on 4-μm tissue sec-tions with a Ventana Bench Mark XT Autostainer (Ventana Medical Systems, Tucson, AZ, USA). Primary anti-bodies included those for CD3 (dilution 1:100; polyclonal; Dako, Glostrup, Denmark), CD4 (RTU; clone CD4-1F6; Novocastra, Leica, Newcastle upon Tyne, UK), CD8 (RTU; clone C8/144B; Dako), CD56 (dilution 1:100; clone CD564; Novocastra), CD30 (dilution 1:50; clone Ber-H2; Dako), CD103 (dilution 1:50; polyclonal; Novus Biolog-icals, Littleton, CO, USA), CD10 (dilution 1:100; clone 56C6; Novocastra), PD-1 (dilution 1:100; clone NAT105; Cell Marque, Rocklin, CA, USA), granzyme B (dilution 1:50; clone GrB-7; Dako), TIA-1 (dilution 1:100; clone 2G9A10F5; Beckman Coulter, Marseille, France), ALK (dilu-tion 1:50; clone ALK1; Dako), and Ki-67 (dilution 1:150; clone MIB-1; Dako). Pos-itivity for these markers was defined as expression in ≥ 30% of tumor cells [11]. EBER in situ hybrid-ization was performed with digoxigenin-labeled probes (Novocastra) and a Ventana Bench Mark XT Autostainer.

Table S1. First-line treatment of primary gastrointestinal T/NK cell lymphoma.

Treat-ment	ENKTL (n=9)	MEITL (n=9)	ITCL (n=6)	ALCL, ALK- (n=4)	ALCL, ALK+ (n=1)	Total (n=29)
CHOP	4 [a]	5 [b]	6	3	1	17 (58.6)
IMVP-16PL	3	2	0	1	0	6 (20.7)
Other	2 [c]	2 [d]	0	0	0	6 (20.7)

ALCL, ALK-; anaplastic large cell lymphoma, ALK-negative, ALCL, ALK+; anaplastic large cell lymphoma, ALK-positive, CHOP; cyclophosphamide, doxorubicin hydrochloride, vincristine sulfate, and prednisone, ENKTL; extran-odal NK/T cell lymphoma, nasal type, IMVP-16PL; ifosfamide, methotrexate, etoposide, cyclophosphamide, and dexamethasone, ITCL, NOS; intestinal T-cell lymphoma, not otherwise specified, MEITL; monomorphic epitheliotropic intestinal T-cell lymphoma. [a] Three patients were treated with a regimen supplemented with etoposide with CHOP (EPOCH). [b] A patient was treated with a regimen supplemented with etoposide with CHOP (EPOCH). [c] SMILE (dexamethasone, methotrexate, ifosfamide, l-asparaginase, and etoposide). [d] One patient was treated with vincristine with dexamethasone and the other received hyper-CVAD (cyclophosphamide, vincristine, doxorubicin, dexamethasone, with methotrexate and cytarabine).

Table S2. The results of whole blood EBV quantitative PCR test in patient with primary gastrointestinal T/NK cell lymphoma.

Results	ENKTL (n=8)	MEITL (n=9)	ITCL (n=6)	ALCL, ALK- (n=3)	ALCL, ALK+ (n=1)	Total (n=26)
Positive	3 (37.5)	3 (33.3)	1 (16.7)	0 (0)	0 (0)	7 (36.8)
Titer	206,825	25,450	7,708			

(Copies/mL)	134,458	11,087				
	182,376	740				
Negative	5 (62.5)	6 (66.7)	5 (83.3)	2 (100)	1 (100)	19 (73.2)

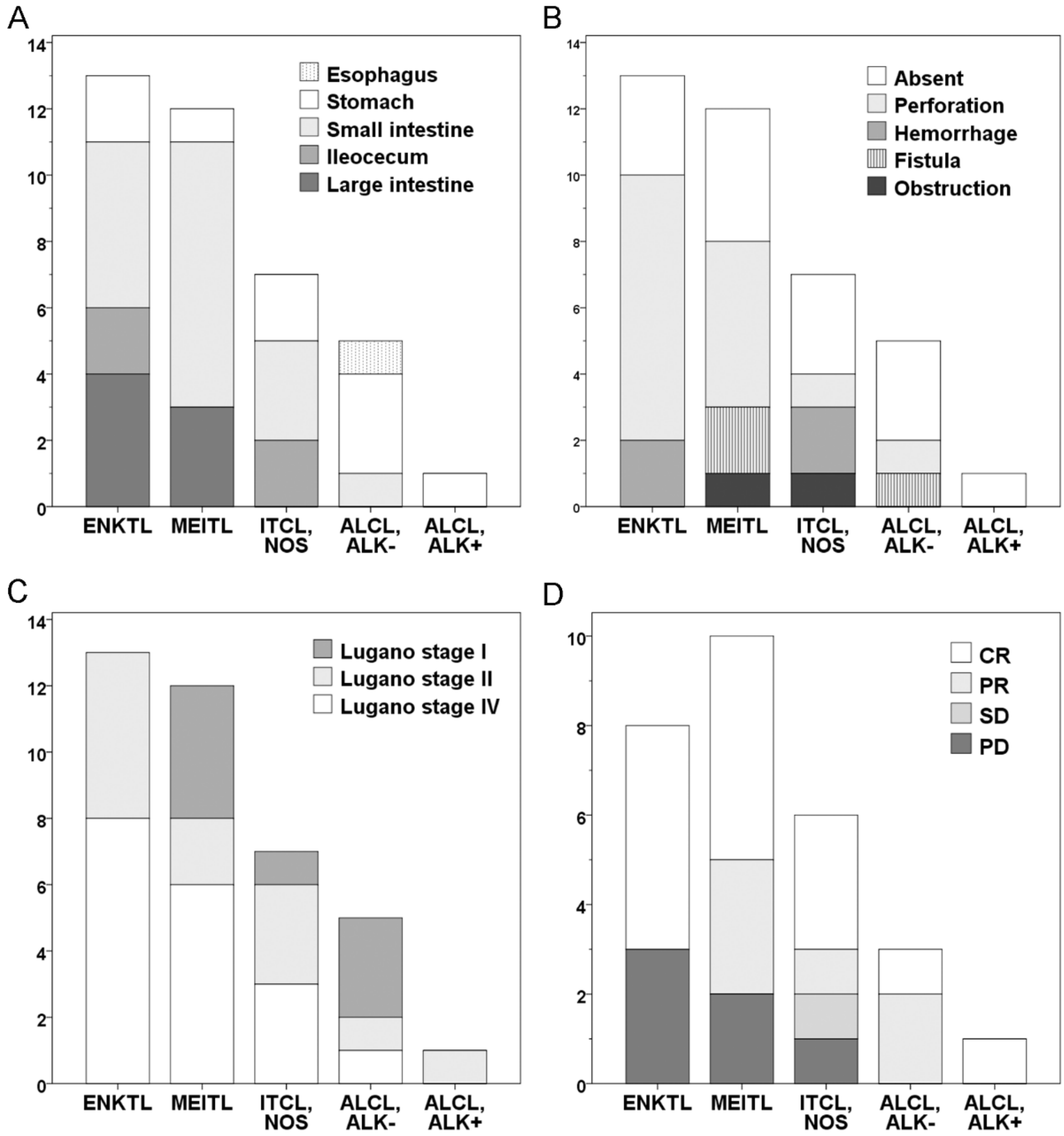

Figure S1. Comparison of clinical features of primary gastrointestinal T/NK cell lymphoma sub-types. Location (A), gastrointestinal complication (B), Lugano stage (C), and initial treatment response (D). (Abbreviations: ALCL, ALK-, anaplastic large cell lymphoma, ALK-negative; ALCL, ALK+, anaplastic large cell lymphoma, ALK-positive; ENKTL, extranodal NK/T cell lymphoma, nasal type; ITCL, NOS, intestinal T-cell lymphoma, not otherwise specified; MEITL, monomorphic epitheliotropic intestinal T-cell lymphoma).

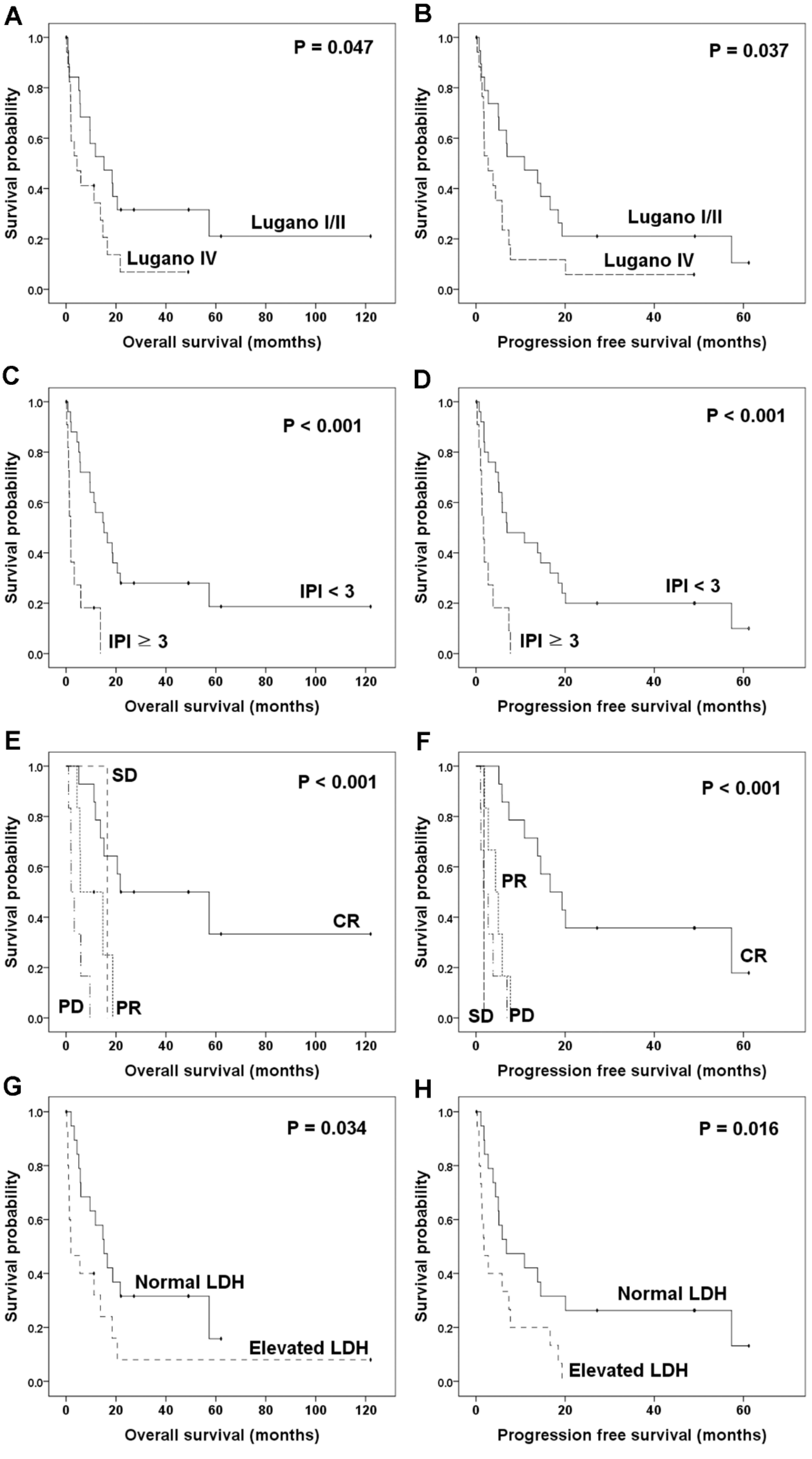

Figure S2. Kaplan-Meier survival curves according to clinicopathological parameters. Overall survival (A, C, E, and G) and progression free survival (B, D, F, and H) based on Lugano stage (A and B), IPI score (C and D), initial treatment response (E and F), and serum LDH level (G and H).
